# Imide Polymers with Bipolar-Type Redox-Active Centers for High-Performance Aqueous Zinc Ion Battery Cathodes and Electrochromic Materials

**DOI:** 10.3390/ijms26083838

**Published:** 2025-04-18

**Authors:** Zixuan Liu, Yan Li, Binhua Mei, Jiaxue Liu, Haijun Niu, Yanjun Hou

**Affiliations:** 1 Key Laboratory of Chemistry, Chemical Engineering and Materials, High-Quality Technology Conversion, Heilongjiang Province & School of Chemistry and Chemical Engineering, Heilongjiang University, Harbin 150086, China; 2221412@s.hlju.edu.cn (Z.L.); 2221436@s.hlju.edu.cn (Y.L.); 2221426@s.hlju.edu.cn (B.M.); 2231502@s.hlju.edu.cn (J.L.); 2 Key Laboratory of Functional Inorganic Material Chemistry, Ministry of Education & Department of Macromolecular Science and Engineering, School of Chemistry and Chemical Engineering, Heilongjiang University, Harbin 150086, China; haijunniu@hotmail.com

**Keywords:** aqueous zinc-ion battery, bipolar-type organic cathode, electrochromic, bifunctional

## Abstract

Aqueous zinc-ion batteries (AZIBs) have attracted interest for their low cost and environmental friendliness. Two bipolar organic materials with different degrees of conjugation, pPMQT and pNTQT, were rationally designed and synthesized as cathode candidates for AZIBs based on 4,4′-diaminotriphenylamine (TPA), 2,7-diaminoanthraquinone (AQ), and two anhydrides. This molecular design features an increased conjugation and electron cloud density, thereby improving charge transport kinetics, specific capacity, and cycling stability. In comparison with pPMQ and pNTQ (n-type), pPMQT and pNTQT demonstrate better electrochemical characteristics. In this work, pNTQT shows outstanding performance. It exhibits an initial capacity of 349.79 mAh g^−1^ at 0.1 A g^−1^ and retains a specific capacity of 190.25 mAh g^−1^ (87.6%) after 5000 cycles at 5 A g^−1^. In comparison, pNTQ demonstrates a specific capacity of only 207.55 mAh g^−1^ at 0.1 A g^−1^, and after 5000 cycles at 5 A g^−1^, its capacity retention rate is only 81.2%. At the same time, both pPMQT and pNTQT polymer films demonstrate attractive electrochromic (EC) properties, displaying reversible color transitions from yellow to dark blue in the UV–visible spectrum. This work lays the foundation for the further development of triphenylamine-based polyimide materials for application in AZIBs and electrochromism.

## 1. Introduction

The demand for global energy is progressively increasing due to the rapid development of human society and the economy [1,2,3]. The main traditional fossil energy sources are coal, oil, and natural gas; however, their reserves are limited and their over-exploitation will lead to significant degradation of the environment. Consequently, it is imperative to research sustainable green energy sources. The transition from the fossil fuel era to the renewable green energy era is both inevitable and imperative [4,5,6,7,8,9]. Aqueous metal-ion batteries have provided us with an unprecedented, convenient renewable green energy storage technology option, replacing polluting fossil fuels. In contrast to organic electrolytes, aqueous batteries employ safe aqueous electrolytes. Many rechargeable aqueous metal-ion batteries, such as those based on sodium, potassium, aluminum, calcium, and zinc ions, have been developed to date [10,11,12,13,14,15]. Among them, AZIBs have received much attention from researchers as a rising star in the green energy field due to their unique advantages, such as their low cost, high electrical conductivity, and high theoretical capacity [16,17,18,19,20]. Recent advancements in research have highlighted the potential applications of various organic materials and their composites in AZIBs. This is primarily attributable to the intrinsic benefits of organic chemicals compared with inorganic ones, such as reduced prices, renewability, structural adaptability, and ecological sustainability [21,22,23]. Calix [4] quinone (C4Q) is a novel benzoquinone-based cathode material for AZIBs with a specific capacity of up to 335 mAh g^−1^, which indicates that quinone organic cathode materials have excellent potential for application in AZIBs. However, their poor electrical conductivity and the high solubility of the discharge products have significantly hindered their further development [24]. It has been widely demonstrated that polymerization allows for the effective building of stable and flexible frameworks for better recyclability [25,26,27,28,29]. Carbonyl materials as positive electrodes are typical n-type materials and mainly store energy at the C=O group active site [30]. n-Type organic cathode materials often exhibit greater specific capacity owing to the greater density of active sites per unit weight. Given, as already mentioned, that the lower operating voltage and the higher solubility of discharge products hinder their further development, a reasonable structural design is essential to enhancing electrical conductivity and operating voltage while preserving a high specific capacity [31,32].

Triphenylamine (TPA) is a prevalent p-type organic electrode material in which the central N atoms initially undergo an oxidation process, during which they lose electrons and turn into cations; the latter then undergo reversible insertion/release with the anions in the electrolyte for energy storage [33]. p-type organic materials exhibit faster ion transfer rates because little bond rearrangement occurs during charging and discharging [31]. However, because they have fewer active sites in their structure, they usually have a lower specific capacity [34]. In comparison with p-Type and n-type organic cathode materials, bipolar-type conducting polymers concurrently exhibit a superior operating voltage, improved electronic conductivity, and exceptional specific capacity [35].

TPA and its derivatives have been extensively studied and applied in the field of EC materials due to their central nitrogen atom having a lone pair of electrons, which are prone to redox reactions that are often accompanied by intense color changes during oxidation. EC refers to the reversible alteration in the optical properties of materials, including their appearance, color, and transparency, induced by an external electric field [36,37,38,39]. Ions are inserted or extracted during the EC process under external electrical stimulation. This behavior resembles the electrochemical redox reactions or ion insertion/dissolution processes observed during battery charging and discharging under applied voltage [40]. Therefore, bifunctional materials with EC and AZIB properties have great potential for development.

Herein, two bipolar-type electrode materials containing quinone polyimides with TPA groups (pPMQT and pNTQT) were prepared as oxidation-active electrode materials for AZIBs and EC materials, using 2,6-diamino anthraquinone, TPA, and two anhydrides with different degrees of conjugation (PMDA or NTCDA) as the raw materials. Compared with anthraquinone polyimides, pPMQT and pNTQT have higher specific capacity, cycling stability, and operating voltage. It is noteworthy that the theoretical capacity of pNTQT, calculated according to the formula provided in the Supplementary Materials, is 488.66 mAh g^−1^. When tested at a current density of 0.1 A g^−1^, pNTQT exhibits an initial specific capacity of 349.97 mAh g^−1^, which is significantly higher than that of pNTQ (207.55 mAh g^−1^) under the same conditions. Even after 5000 cycles at a current density of 5 A g^−1^, it retains a specific capacity (190.25 mAh g^−1^) that is 87.4% of the initial capacity, demonstrating good stability. However, at the same current density and number of cycles, pNTQ only maintains 80.1% of its initial specific capacity. In the EC investigation, the pPMQT and pNTQT films exhibited significant color change from yellow to inky blue, demonstrating outstanding EC properties, including rapid switching time and high coloring efficiency.

## 2. Results and Discussion

### 2.1. Physicochemical Characterizations

**Scheme 1:**
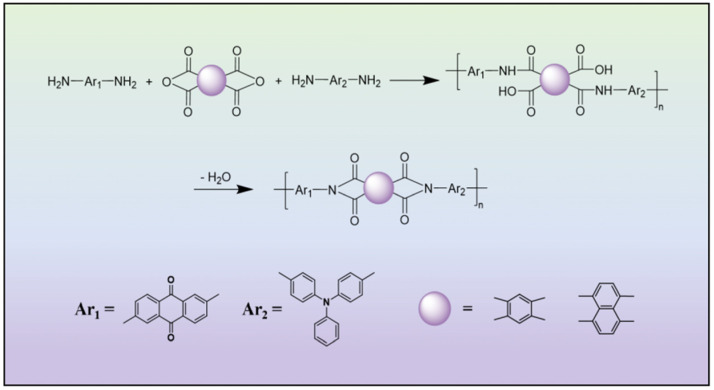
Synthetic routes of pPMQT and pNTQT.

Scheme 1 and Figures S1 and S2 depict the detailed synthetic procedures and molecular structures of the monomer 4,4-diaminotriphenylamine as well as the four polyimide polymers [41]. We utilized a combination of analytical techniques, including NMR, FTIR spectroscopy, UV–vis spectrophotometry, and AFM, to thoroughly characterize the chemical structure and morphology of these polyimide derivatives. The FTIR spectra depicted in Figures S3 and S4 demonstrate the absence of the characteristic peaks associated with the C–O–C stretching vibrations of anhydride groups in the synthesized polymers (for PMDA, at 1236 cm^−1^, and for NTCDA, at 1121 cm^−1^). Conversely, the FTIR spectra of the four polymers exhibit characteristic peaks corresponding to the stretching vibrations of imide C–N–C bonds (for pPMQ and pPMQT, at 1366 cm^−1^, and for pNTQ and pNTQT, at 1326 cm^−1^). This result confirmed the cleavage of C–O–C bonds and the formation of new C–N–C bonds during the synthesis. Additionally, the FTIR spectra exhibit characteristic peaks corresponding to the C=O group of anthraquinone at approximately 1592 cm^−1^ and 1673 cm^−1^. The absence of significant signals of –NH_2_ groups further indicates the successful completion of the polymerization reactions. The latter was further corroborated by the ^1^H NMR spectra of the four polymers, as shown in Figures S5–S9. Notably, the presence of obvious large peaks belonging to the triphenylamine group in pPMQT and pNTQT at 6.8–7.5 ppm and 7.0–7.5 ppm, respectively, further proved the successful incorporation of the triphenylamine group.

According to the thermogravimetric analyses (TGAs) of pPMQT and pNTQT, both show greater thermal stability than pPMQ and pNTQT, as can be seen in Figure S10. pPMQT and pNTQT maintain their structural stability at high temperatures of up to 400 °C, which indicates that they are very safe to be used as rechargeable battery materials.

### 2.2. Quantum Chemistry Calculation

**Figure 1:**
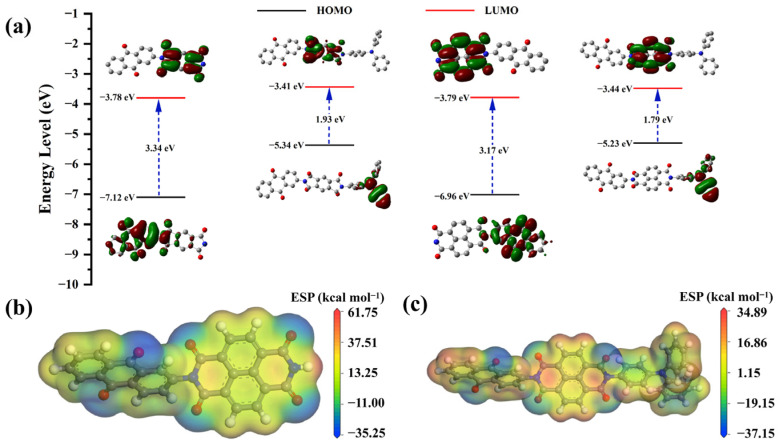
(**a**) The modeling of the HOMO and LUMO of pPMQ, pPMQT, pNTQ, and pNTQT and the electrostatic potential (ESP) of pNTQ (**b**) and pNTQT (**c**).

Polyimide derivatives based on TPA, AQ, and anhydrides are bipolar-type polymers. This kind of organic material exhibits not only excellent specific capacity but also stronger conductivity and a faster electron transfer rate. Density Functional Theory (DFT) was employed to determine the Highest Occupied Molecular Orbital (HOMO) and Lowest Unoccupied Molecular Orbital (LUMO) energy levels of the four polymers. As shown in Figure 1a, the HOMO/LUMO levels of the four polymers are −3.78/−7.12 eV (pPMQ), −3.41/−5.34 eV (pPMQT), −3.79/−6.96 eV (pNTQ), and −3.44/−5.23 eV (pNTQT). From the data, it can be found that pPMQT and pNTQT have higher HOMOs than pPMQ and pNTQ, which is mostly attributed to the fact that the central N atom in the TPA group has a lone pair of electrons, which increases the electron cloud density of the polymers. Due to the elevated degree of conjugation in naphthalene molecules, pNTQT exhibits a lower LUMO value than pPMQT, resulting in a reduced energy gap (Eg) [42].

Their Eg values were calculated to be 3.34 eV (pPMQ), 1.94 eV (pPMQT), 3.17 eV (pNTQ), and 1.79 eV (pNTQT). Polymers with TPA groups exhibit a narrower Eg. This also confirms that the bipolar-type organic material has a better electron transfer rate and stronger conductivity; thus, pPMQT and pNTQT are expected to show better electron storage performance. The UV–vis spectrum and CV reveal that with regard to Egs, pNTQT < pPMQT, aligning with theoretical calculations.

In addition, we calculated and analyzed the molecular electrostatic potential (ESP) of the polymers, where the blue patches indicate areas of elevated electronegativity serving as the primary active sites. Figure 1b,c and Figures S11 and S12 illustrate multiple negative regions surrounding the O atoms in the polymers, indicating their higher electronegativity compared with other regions. Notably, the proximity of the O atoms in pPMQT and pNTQT exhibits a more negative ESP than in pPMQ and pNTQ, which is attributable to the incorporation of the triphenylamine moiety, suggesting that the C=O groups in pPMQT and pNTQT possess an enhanced ability to attract cations. In contrast, the region around the central N atom of the TPA group in pPMQT and pNTQT shows a negative ESP, which indicates that the N atom at the center of TPA in polymers is also the active site for the reaction. Overall, as pPMQT and pNTQT have more active sites, as well as a smaller Eg, they have a higher electron transfer rate and specific capacity.

### 2.3. Electrochemical Analyses

**Figure 2:**
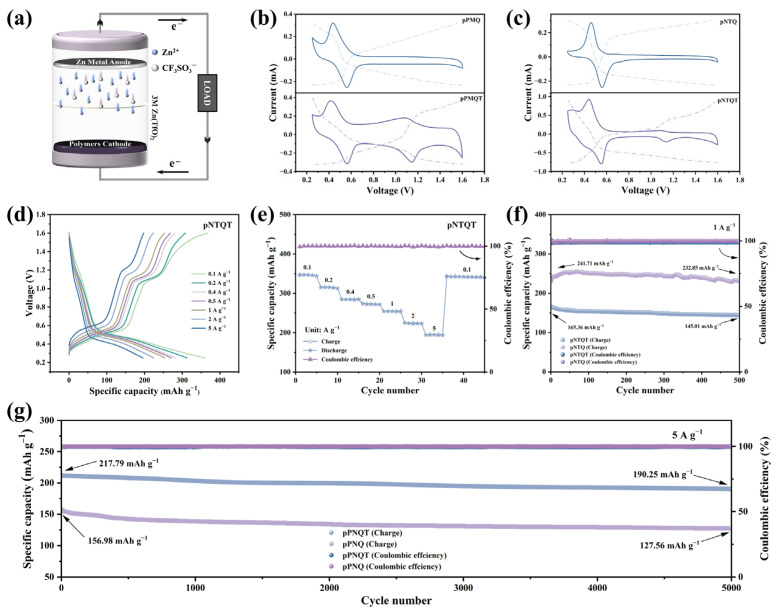
(**a**) Basic diagram of button cell. CV data for pPMQ and pPMQT (**b**), and pNTQ and pNTQT. (**c**) Half cells scanned at 0.2 mV s^−1^ and discharge and charge curves (imaginary line) in 3 mol L^−1^ Zn(TfO)_2_. pNTQT under varying current densities: (**d**) discharge and charge curves and (**e**) rate performance. Comparison of long-term cycling stability of pNTQ and pNTQT (**f**) at 1 A g^−1^ for 500 cycles and (**g**) at 5 A g^−1^ for 5000 cycles.

Subsequently, we created button-types batteries by coating four polymers on stainless-steel mesh as the cathode with a metal zinc anode and evaluated their electrochemical properties by using CV and constant-current charge/discharge (GCD) (Figure 2a). As illustrated in Figure 2b,c, we used CV in the voltage range of 0.25–1.6 V at a scan rate of 0.2 mV s^−1^ to analyze the electrochemical behaviors of the polymers and compared them with the GCD curves (dashed lines). At 0.2 mV s^−1^, pPMQ (0.56/0.32 V) and pNTQ (0.56/0.46 V) exhibit a single pair of distinct redox peaks, which can be attributed to the reaction of C=O in the structure. Notably, in NTCDA-based materials, CV profiles typically exhibit two pairs of redox peaks. However, in our experimental measurements, only a single pair of redox peaks was detected. This deviation is presumably attributable to the close proximity of the two reduction potentials of the NTCDA-based polymer under investigation in the aqueous electrolyte, resulting in the overlap or merging of the corresponding peaks. Furthermore, stacking is observed in the polymer materials, which further blurs the second redox process [43]. In contrast, pPMQT (0.56/0.36 V and 1.15/1.1 V) and pNTQT (0.55/0.44 V and 1.14/1.1 V) show two sets of oxidation/reduction peaks (detailed data are shown in Table S1). This indicates that pPMQT and pNTQT undergo two separate redox processes. The first set of redox peaks, located at 0.56/0.36 V for pPMQT and 0.55/0.44 V for pNTQT, can be attributed to the reversible redox behavior of the C=O in the polymer backbones. Furthermore, the second set of redox peaks, observed at 1.15/1.10 V (pPMQT) and 1.14/1.10 V (pNTQT), is likely associated with the redox of the N atoms of the TPA centers. The dashed line part of the figure shows the GCD curves of the polymers, and by overlapping and comparing them with the CV findings, the results show that the plateaus of the GCD curves of the four polymers coincide with the voltages of the redox peaks of the CV curves. Moreover, at the same scan rate, pNTQT has a greater CV area than the other polymers, and these characteristics align with the GCD results, indicating that pNTQT possesses higher specific capacity and demonstrates superior electrochemical performance.

In a further evaluation of their properties, the specific capacity and stability of the polymers were assessed. The specific capacity values of the four polymer cathode materials at various current densities are presented in Figure 2d,e and Figures S13–S15. As shown, pNTQ exhibits specific capacities of 207, 182, 173, 169, 162, 154, and 144 mAh g^−1^ at the current densities of 0.1, 0.2, 0.4, 0.5, 1, 2, and 5 A g^−1^, respectively. In contrast, after the incorporation of triphenylamine groups, the synthesized pNTQT achieves values of 350, 312, 282, 269, 253, 224, and 198 mAh g^−1^, respectively, showing a significant improvement in this property with respect to pNTQ. It is noteworthy that at 0.1 A g^−1^, pNTQT delivers an initial specific capacity of 349.79 mAh g^−1^, significantly greater than that of pNTQ (207.55 mAh g^−1^). Additionally, when the current is reverted to 0.1 A g^−1^, the reversible specific capacity of pNTQ decreases to 196 mAh g^−1^, i.e., 94.6% of the initial specific capacity, and there is a significant drop after 10 cycles. In contrast, pNTQT maintains a reversible specific capacity of 347 mAh g^−1^, corresponding to 98.8% of the initial specific capacity, and negligible capacity loss after the same number of cycles, demonstrating improved stability. Similarly, pPMQT exhibits markedly superior rate performance compared with pPMQ. Both pPMQT and pNTQT exhibit significantly higher specific capacity than pPMQ and pNTQ at various current densities. This effect can be attributed to the incorporation of TPA groups, which enables the formation of p/n bipolar materials. This doping increases the operating voltage of pNTQT and pPMQT while increasing the degree of conjugation within the systems, thereby enhancing their battery performance. Secondly, the examination of the polymer ESP indicates that pPMQT and pNTQT have more active sites, as well as a more negative ESP surrounding the O atoms, which enables them to capture a higher quantity of cations. Therefore, compared with the n-type cathode materials (pPMQ and pNTQ), pPMQT and pNTQT combine the advantages of higher operating voltage and superior specific capacity. Additionally, pNTQT exhibits better specific capacity than pPMQT, which is attributable to the bigger aromatic conjugated structure and higher density of active centers in NTCDA relative to PMDA, which collectively improve the specific capacity of the polymers.

Cycling stability is an important factor in measuring battery performance, so we evaluated this property in the polymers at 1 A g^−1^. Among them, pNTQT has an initial specific capacity of 241.71 mAh g^−1^, which, after 500 cycles, decreases to 232.85 mAh g^−1^, with a retention rate of 96.3% (Figure 2f). However, the pNTQ cathode material exhibits a notable capacity decay after 500 cycles at the same current density, retaining only 87.35% of its initial specific capacity. In comparison, pPMQT demonstrates greater cycling stability, maintaining 90.4% of its initial capacity, than pPMQ (86.7%) under the same current density conditions (Figure S16). Figure 2g displays the long-term cycling performance of the polymers at 5 A g^−1^. As shown in the figure, the pNTQ cathode material exhibits an obvious decline in specific capacity before reaching 500 cycles, followed by a gradual decrease. After 5000 cycles, the specific capacity is 127.56 mAh g^−1^, which is 81.2% of the initial specific capacity. In contrast, pNTQT, with a longer conjugated structure, shows almost no capacity loss after 500 cycles at a current density of 5 A g^−1^, with a retention rate of 87.6% after 5000 cycles. These results show that the pNTQT material demonstrates outstanding cycling stability. pPMQ and pPMQT also showed the same results (Figure S17). These findings suggest that the incorporation of triphenylamine increases the degree of conjugation within the structure, effectively enhancing the battery operating voltage and introducing additional active sites. As a result, this strategy significantly improves the specific capacity and cycling stability of the cathode materials. Moreover, the larger conjugated structure of NTCDA, compared with PMDA, further contributes to the increase in the material’s specific capacity and stability.

### 2.4. Analysis of Redox Kinetics

**Figure 3:**
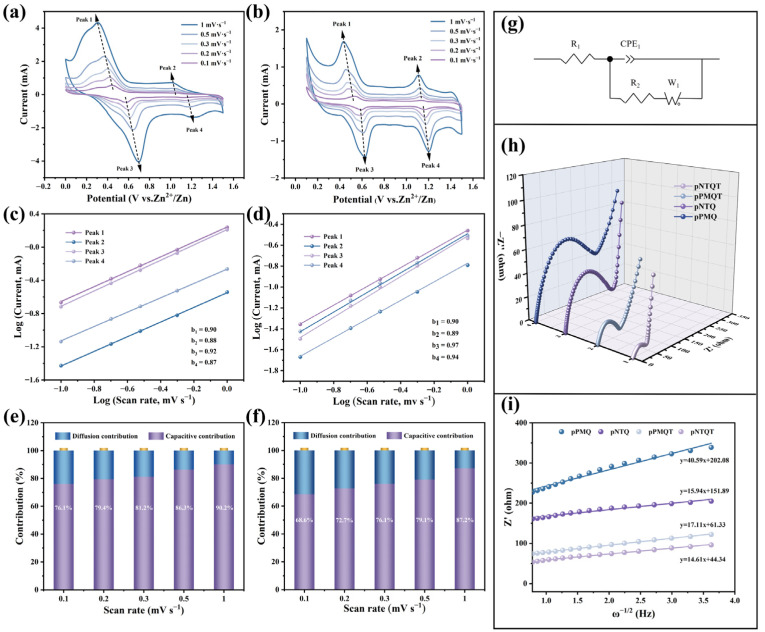
The CV curves of pNTQT (**a**) and pPMQT (**b**) at different scan rates. The fitted curves of the calculated b values of pNTQT (**c**) and pPMQT (**d**). The contributions of capacitive and diffusion control of pNTQT (**e**) and pPMQT (**f**) at ranging scan rates. (**h**) The impedance spectra of pPMQ, pNTQ, pPMQT, and pNTQT collected at open-circuit voltage ((**g**) middle: the equivalent circuit diagram). (**i**) The relation between the Zre and ω−1/2 of pPMQ, pNTQ, pPMQT, and pNTQT at a low frequency.

An electrochemical workstation was used to investigate the kinetic performance of the polymer materials. The voltage range for pPMQT and pNTQT was 0–1.5 V, while that for pPMQ and pNTQ was restricted to 0–0.9 V, as their oxidation/reduction peaks are only observed within this range. As shown in Figure 3a,b and Figures S18a and S19a, we used CV to determine the redox kinetics of the four polymers at varying scan rates (0.1, 0.2, 0.3, 0.5, and 1 mV s^−1^). As the scan rate increases, the shape of the CV curve remains largely consistent, while the area of the curve increases. However, a slight shift in the oxidation and reduction peaks is observed towards higher and lower potentials, respectively, owing to the polarization effects induced by the higher scan rates. The following equation delineates the correlation between peak current (*i*) and scan rate (*v*) [44]:

*i* = *av^b^* (1)

log *i* = log *a* + *b* log *v* (2)

where *i* is the peak current of the CV curve, *v* is the scan rate, and *a* and *b* are adjustable parameters. The value of *b* can be calculated from the slope of the fitted curve of log *i* and log *v*. Diffusion control occurs when the value of *b* is near 0.5. When *b* is near 1, the process is capacitance-controlled. pPMQT and pNTQT had *b* values of 0.90/0.97 and 0.89/0.94 (Figure 3c), and 0.90/0.92 and 0.88/0.87 (Figure 3d), respectively, which are all closer to 1. This result suggests that the redox behaviors of the polymers predominantly display capacitance control. The *b* values of pPMQ and pNTQ are also near 1 (Figures S18b and S19b). The following equation was used to further determine their individual contribution ratios [45]:

*i_v_* = *k*_1_*v* + *k*_2_*v*^1/2^ (3)

where *i_v_* is the total current and *k*_1_*v* and *k*_2_*v*^1/2^ are the proportions of capacitance and diffusion control in the total current, respectively. Figure S20 depicts the capacitive contribution (blue region) and the diffusion control process contribution (white region) to the CV current of pNTQT at 0.5 mV s^−1^; moreover, its capacitive contribution increases from 76.1% to 90.2% as the scan rate increases (Figure 3e). The capacitive contributions of pPMQT (Figure 3f), pPMQ (Figure S22), and pNTQ (Figure S23) increased from 68.6%, 56.1%, and 73.3% to 87.2%, 79.7%, and 87.4%, respectively, with the increase in the scan rate.

The data revealed that the two polymers containing TPA groups exhibited accelerated redox kinetics, with pNTQT demonstrating the greatest rate performance and best kinetic characteristics. Furthermore, electrochemical impedance spectroscopy (EIS) was employed to investigate the kinetic behaviors of the polymers. Figure 3g illustrates that the EIS plot features a semicircle in the high-frequency region and diagonal lines in the low-frequency region, where a reduced semicircle radius signifies lower resistance, enhanced conductivity, and a higher electron transfer rate. Based on the fitted and equivalent circuits, the semicircular diameter of pNTQT is significantly smaller than that of the other polymers, indicating lower charge transfer impedance. Therefore, pNTQT has a higher charge transfer rate as an organic cathode material. Figure 3h depicts the linear relationship between *Z_re_* and *ω*^−1/2^ for the polymer cathode organic materials. The results show that pNTQT has the largest diffusion coefficient, with a slope value of 14.61 [46]. These results are consistent with the conclusions of DFT, indicating that the doping of triphenylamine significantly enhances the electron transfer rate.

### 2.5. Electrochemical Reaction Mechanism

**Figure 4:**
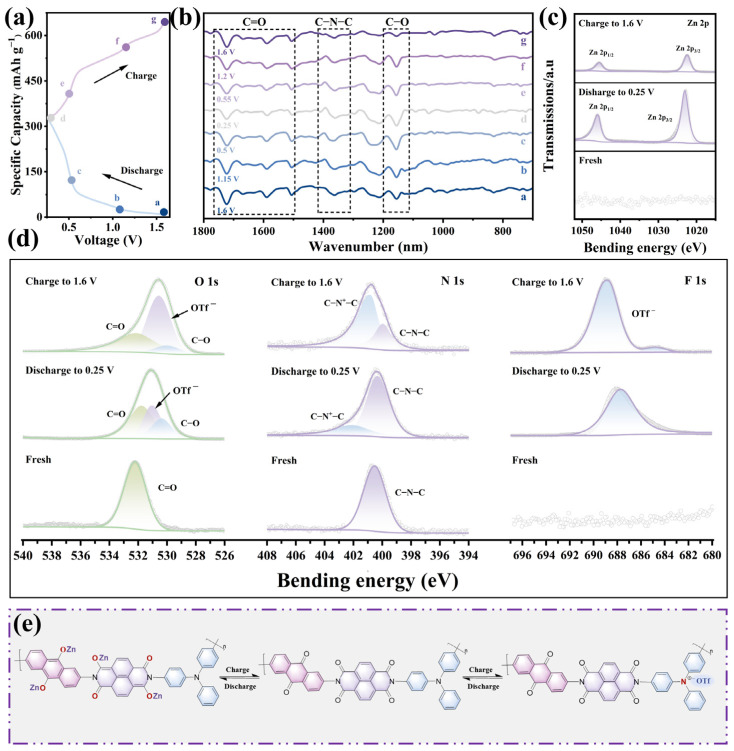
(**a**) Charge/discharge voltage profiles of pNTQT cathode. (**b**) Ex situ FTIR of pNTQT cathode during charge/discharge processes. Ex situ XPS spectra of pNTQT cathode in pristine, fully charged (1.6 V) and fully discharged (0.25 V) states of (**c**) Zn 2p and (**d**) O 1s, N 1s, and F 1s spectra (left, middle, and right). (**e**) Schematic electrochemical reaction mechanism of pNTQT in aqueous Zn(OTf)_2_ electrolyte.

Characterization analyses using FTIR and XPS techniques were conducted to examine the storage mechanism of the polymer cathodes under various charging and discharging conditions. Figure 4a and Figure 4b show the charge/discharge voltage profiles of the pNTQT cathode and its ex situ FTIR spectra in different charge/discharge states, respectively. The results show that the characteristic peaks of the C=O groups located at 1500–1750 cm^−1^ decreased significantly during the discharge process, while the C–O peaks at 1155 cm^−1^ were gradually enhanced in comparison. The peaks of these bonds gradually recovered during the subsequent charging process. This behavior is primarily ascribed to the C=O, serving as the active redox center for Zn^2+^, with which it undergoes reversible reactions, resulting in the interconversion of C=O and C–O. In addition, the peak of the C–N–C group located around 1367 cm^−1^ was enhanced during the discharge process and weakened again when charged. It could thus be suggested that the N atom of TPA lost electrons and was oxidized to N^+^ when charged and combined with the anions (CF_3_SO_3_^−^) in the electrolyte. Conversely, when the voltage was discharged to 0.25 V, the CF_3_SO_3_^−^ anions were extracted, and the C–N–C peak intensity became stronger. The aforementioned analyses indicate that both C=O and C–N–C are active sites. Additionally, we conducted an ex situ XPS analysis of pNTQT in various charging and discharging stages to confirm the bond changes. As shown in Figure 4c,d, during the discharge process (fresh→D 0.25 V), the intensity of C=O (532.33 eV) decreased significantly, accompanied by a gradual C-O (531.69 eV) increase, with the emergence of the OTf^−^ peak [47,48]. Moreover, the trend of C–N^+^–C (402.21 eV)/C–N–C (400.58 eV) was similar to that of C=O/C–O. Subsequently, when charging to 1.6 V (D 0.25 V→C 1.6 V), the intensity of C=O and C–N–C was restored [49]. This regularity in XPS is similar to the trends in IR spectra and reveals the transformations of C=O/C–O and C–N–C/C–N^+^–C. The analysis of Zn 2p and F 1s in XPS revealed that during charging, the intensity of F increased, while the peak area of Zn decreased. As the voltage decreased from 1.6 V to 0.25 V, the F peak diminished, and the intensity of Zn increased. It is worth noting that a portion of CF_3_SO_3_^−^ does not completely dissociate from Zn^2+^ during the storage of Zn^2+^. As a result, the peaks associated with CF_3_SO_3_^−^ do not fully disappear during the discharge process but are diminished. This result further substantiates the presence of two distinct energy storage mechanisms in the pNTQT cathode [50].

**Figure 5:**
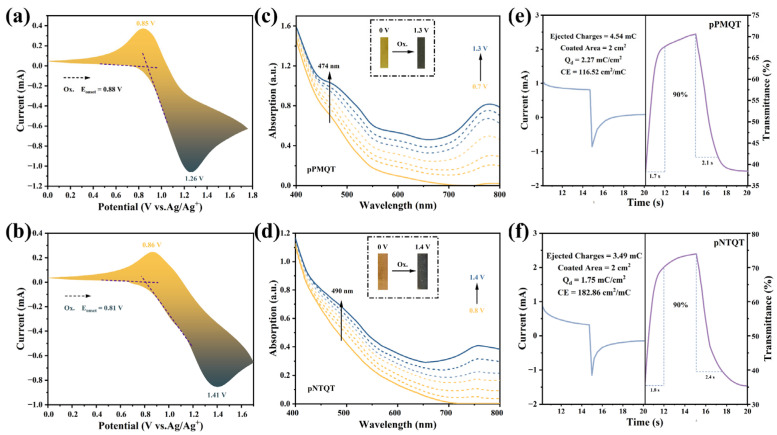
CV curves of pPMQT (**a**) and pNTQT (**b**) on ITO-coated glass substrate in 0.1 mol L^−1^ TBAP/ACN solution at 50 mV s^−1^ scan rate. Absorption spectra and photographs (inset) of pPMQT (**c**) and pNTQT (**d**). Dynamic changes in transmittance and current with pulse width of 10 s and optical switching for pPMQT (**e**) and pNTQT (**f**).

Figure 5e depicts the more detailed electrochemical reaction mechanism of pNTQT, which may be categorized into four distinct stages of the redox process. Here, it is worth noting that based on the literature review and the corresponding formulas (refer to the Supplementary Materials), we predict that only two C=O groups in the NTCDA group of pNTQT are involved in energy storage [43,51,52,53]. During the initial charging process, pNTQT loses electrons from the pristine state and undergoes oxidation to form C–N^+^–C, where CF_3_SO_3_^−^ anions migrate to balance the charge. Subsequently, pNTQT undergoes a continuous reduction reaction in the next two stages. During the first discharging process, C–N^+^–C acquires electrons to initially form C–N–C, accompanied by anion extraction. In the next discharging process, C=O also obtains electrons and reduces to C–O, simultaneously combining with Zn^2+^ to achieve a fully reduced state. The last stage is the restoration of pNTQT from its fully reduced form to its initial state through the loss of electrons and the dissolution of Zn^2+^ during the subsequent charging process. In summary, the initial two steps of the pNTQT redox process relate to the redox activity of the N atom in TPA (p-type doping), and the last two stages are associated with interconversion between C=O and C–O (n-type doping). The storage mechanisms of the other polymers were also tested: pPMQT exhibited the same mechanism as pNTQT (Figure S24). pPMQ and pNTQ exhibited the same storage mechanism, i.e., C=O as the active site of Zn^2+^ and energy storage by C=O/C–O interconversion, which is typical of n-type cathode materials (Figures S25 and S26). The specific XPS atomic concentration data can be found in Table S2.

### 2.6. Electrochromic Properties of pPMQT and pNTQT

As illustrated in Figure S27, the UV–vis spectra of pPMQT and pNTQT in NMP solutions exhibit an absorption peak near 450 nm, which is primarily attributed to π–π* transitions. Further, we performed CV and spectroelectrochemistry studies in 0.1 mol L^−1^ TBAPF6/Propylene Carbonate (PC) solution and investigated the EC properties of pPMQT and pNTQT. Here, ITO glass coated with pPMQT or pNTQT served as the working electrode, and Ag wires and Pt flakes served as the reference and counter electrodes, respectively, to construct a three-electrode system for evaluating the electrochromic properties of the polymers. As depicted in Figure 5a,b, showing the CV data of pPMQT and pNTQT, both exhibit a pair of redox peaks, at 1.26/0.85 V and 1.41/0.86 V, respectively, which are due to the redox reaction of the TPA unit. However, the varying molecule configurations resulted in slightly variable redox potentials. The E_onset_, E_electro_, and E_quantun_ energy level data are shown in Table S2.

Figure 5c,d show that the pPMQT and pNTQT polymer films in the neutral state exhibit yellow and orange colors, respectively. As the voltage was increased to 1.5 V and 1.4 V, the absorption peaks located at 474 nm (pPMQT) and 490 nm (pNTQT), respectively, were significantly enhanced, accompanied by the emergence of a new broad band around 750 nm, which gradually increased in intensity. This phenomenon is attributed to the complete oxidation of the polymers, leading to the formation of di-cations. Furthermore, the color of pPMQT transitioned from yellow to dark blue, while that of pNTQT shifted from orange to dark blue. The response time and CE are important parameters that affect the performance of a polymer’s EC, where the former includes the coloring time (t_c_) and the bleaching time (t_b_) [54]. CE is defined as the change in the optical density of a polymer during the injection (or extraction) of a unit charge [55] We integrated the time–current approach with UV–vis spectrophotometry to determine the response time and CE of the polymers. As shown in Figure 5e,f, after applying a voltage, the polymer film changes from the neutral state to the oxidation state, and the maximum transmittance values of the pPMQT and pNTQT polymers change. When the pulse width is 10 s, the ΔT changes of pPMQT at 474 nm and pNTQT at 490 nm are 32.20% and 38.49%, respectively. It is evident that the optical contrast of pNTQT at a 10 s pulse width is higher than that of pPMQT. All the polymers have shorter response times (pPMQT: t_c_ = 1.7 s, t_b_ = 2.1 s; pNTQT: t_c_ = 1.8 s, t_b_ = 2.4 s). The specific data of the spectroelectrochemistry analyses, CE, tb, and tc of the polymer films are shown in Table S4.

In addition, we examined the surface morphology, as well as roughness, of the polymer films by using atomic force microscopy (AFM). As shown in Figure S28, the polymer films are uniformly distributed and present large roughness (R_q_) values of 0.828 nm for pPMQT and 1.25 nm for pNTQT; i.e., the pNTQT film has a rougher surface, which not only provides more channels for the transportation of electrolyte ions but also leaves enough space for the embedding and extraction of ions [56].

## 3. Materials and Methods

### 3.1. Materials

All chemical reagents and basic materials used in this study were purchased and were used without further processing or purification.

### 3.2. Synthesis

The explicit synthesis procedures for pPMQ (poly 2-(9,10-dioxo-dihydroanthracenyl)-6-methylpyrrolo [3,4-f]isoindole-1,3,5,7-tetraone), pNTQ (poly 2-(9,10-dioxo-dihydroanthracenyl)-7-methylbenzo[lmn][3,8]phenanthroline−1,3,6,8-tetraone), pPMQT (poly 2-(9,10-dioxo-dihydroanthracenyl)-6-(4-(diphenylamino)phenyl)pyrrolo [3,4-f]isoindole-1,3,5,7-tetraone), and pNTQT (poly 2-(9,10-dioxo-dihydroanthracenyl)-7-(4-(diphenylamino)phenyl)benzo[lmn][3,8]phenanthroline-1,3,6,8-tetraone) are described in Scheme 1 and Figure S2. Detailed synthetic steps for the monomer 4,4′-diaminotriphenylamine are shown in the Supplementary Materials and Figure S1.

#### 3.2.1. Synthesis of pPMQ and pNTQ

pPMQ and pNTQ were synthesized by using two-step methods. First, 2.5 mmol AQ (0.596 g) was added to 5 mL of DMAc and stirred until dissolution. Then, 2.5 mmol PMDA (0.545 g) or NTCDA (0.67 g) was gradually added to the solution in three portions. The mixture formed a poly(amic acid) solution after being stirred for 2 h at room temperature. A mixture of 1 mL of DMAc and 0.5 mL of pyridine dissolved in DMAc was then added to the poly(amic acid) solution and kept at room temperature with constant stirring for 1 h. The temperature was then increased to 120 °C and maintained for 48 h. Upon completion of the reaction, the resulting solid–liquid mixture was transferred into cold methanol, followed by stirring and filtration to yield a brown solid. The solid was then subjected to Soxhlet extraction with a 2:1 mixture of methanol and dimethylacetamide (DMF).

#### 3.2.2. Synthesis of pPMQT and pNTQT

pPMQT and pNTQT were prepared by using a procedure similar to that for pPMQ and pNTQ, as indicated in Scheme 1. The difference is that the first step involved weighing 4,4-diamino triphenylamine (0.688 g) and AQ (0.596 g), adding them to 5 mL of DMAc, and stirring to dissolve them. The rest of the procedure was the same.

#### 3.2.3. Electrochemical Measurements

Button-type cells were fabricated from a positive- and negative-electrode shell, a positive electrode with active material attached, a glass fiber membrane impregnated with 3 mol L^−1^ Zn(TfO)_2_, and a zinc-negative electrode. The cathode material was obtained by mixing the active materials (pPMQ, pNTQ, pPMQT, and pNTQT), Cochin Black, and glues (polytetrafluoroethylene, PTEE) in ethanol in a ratio of 7:2:1; then, we coated the resulting slurry on a stainless steel mesh and finally dried it under vacuum conditions at 80 °C.

#### 3.2.4. Polymer Films

ITO was first cleaned with acetone, ethanol, and deionized water 1–2 times, and the polymer (10 mg) was dissolved in NMP (1 mL) to form a solution and then evenly spread on the cleaned ITO. Finally, it was dried under vacuum conditions to obtain ITO covered with a uniform polymer film.

### 3.3. Characterizations

^1^H NMR: Deuterated dimethyl sulfoxide (DMSO-d_6_) and deuterated chloroform (CDCl_3_) were used as solvents, and a Bruker AC−400 MHz spectrometer was used for testing.

FTIR: The tests were performed with a PerkinElmer Spectrum 100 spectrometer, which covers a range from 400 to 4000 cm^−1^.

UV–vis: NMP was used as the solvent, and a Shimadzu UV−3600 spectrophotometer was used for testing.

Thermogravimetric analyses (TGAs): a S3PerkinElmer Pyris 6 TGA instrument was used.

Atomic force microscopy (AFM): We used a S3PerkinElmer Pyris 6 TGA instrument.

Density Functional Theory (DFT): Calculations were performed by using Gaussian 03 software on an SGI Origin 350 server and Baker’s Three Parameter Gradient Corrected Functional Theory (B3LYP).

Molecular electrostatic potential (ESP): This was calculated using Materials Studio 2017 software.

Electrochemical impedance spectroscopy (EIS) and cyclic voltammetry (CV): The analyses were performed with a CHI 670D electrochemical workstation.

A CT-ZWJ-4S-T-1U high-performance battery testing system by NEWARE was used to test the dynamics of the AZIB.

### 3.4. Calculation Methods

#### 3.4.1. Theoretical Specific Capacity

The theoretical specific capacity (Q_Theroy_) of the cathode material can be quantitatively predicted based on the following equation [57]:

$$Q_{Theroy}=\frac{nF}{3.6\mu }$$

Here, *n* denotes the total number of electrons that can be transferred per active site within a single repeating unit of the polymer. *F* is the Faraday constant, representing the electric charge carried by one mole of electrons, typically taken as 96,485 C·mol^−1^. *M* refers to the molar mass of one repeating unit of the polymer material, expressed in g·mol^−1^. The factor 3.6 is a unit conversion coefficient used to convert charge from coulombs (C) to milliampere-hours (mAh).

#### 3.4.2. Calculation of Electron Transfer Number

For reversible or quasi-reversible redox processes, the peak current of the CV curve is related to the scan rate, diffusion coefficient, electrode area, and the number of electrons transferred. According to the Randles–Sevcik equation:

$$i_{p}=(2.69\times 10^{5})\cdot n^{3/2}\cdot A\cdot C\cdot D^{1/2}\cdot v^{1/2}$$

Here, *i_p_* denotes the peak current in the CV curve, with units of amperes (A); *n* is the number of electrons transferred; *A* represents the surface area of the working electrode (cm^2^); *C* is the concentration of the electroactive species (mol cm^−3^); *D* is the diffusion coefficient (cm^2^ s^−1^); and *v* is the scan rate (V s^−1^). Based on the Randles–Sevcik equation, *n* can be derived through the following transformation [58]:

$$n={\left(\frac{i_{p}}{2.69\times 10^{5}\cdot A\cdot D^{1/2}\cdot C\cdot v^{1/2}}\right)}^{2/3}$$

## 4. Conclusions

In this work, we synthesized two imide polymers with bipolar redox-active sites, pPMQT and pNTQT, through the polymerization of AQ, TPA, and two kinds of acid anhydrides with different conjugation degrees. The incorporation of triphenylamine increases the conjugation of the system while simultaneously increasing the electron cloud density of the polymers. As a result, this design strategy significantly enhances the kinetics, specific capacity, and cycling stability of the polymers. Furthermore, the results show that the polymer films of pPMQT and pNTQT have great EC properties and clear discoloration. pPMQT and pNTQT cathodes have initial specific capacities of 217.13 and 349.79 mAh g^−1^ at 0.1 A g^−1^, respectively. Notably, pNTQT exhibits a specific capacity of 190.25 mAh g^−1^ after 5000 cycles at 5 A g^−1^, with a retention rate of 87.4%. FTIR and XPS analyses verified that the pPMQT and pNTQT cathodes mainly store energy through reversible conversions of C=O/C–O and C–N–C/C–N^+^–C. The polymer films produced by uniformly depositing the NMP solution of the polymers onto ITO glass can reversibly transition from yellow to dark blue upon the application of voltage. pPMQT and pNTQT have better EC properties with shorter response times. These findings indicate that the imide polymers containing TPA groups designed and prepared in this study hold significant potential for the development of AZIBs and EC bifunctional materials with outstanding performance.

## Data Availability

The original contributions presented in this study are included in the article/Supplementary Materials. Further inquiries can be directed to the corresponding authors.

## Supplementary Materials

The following supporting information can be downloaded at: https://www.mdpi.com/article/10.3390/ijms26083838/s1.
