# Supplementary material for: Imide Polymers with Bipolar-Type Redox-Active Centers for High-Performance Aqueous Zinc Ion Battery Cathodes and Electrochromic Materials
Source: Int J Mol Sci. 2025 Apr 18;26(8):3838. doi: 10.3390/ijms26083838 (PMC12027605; doi:10.3390/ijms26083838)
Supplement: Supplementary file 1 [file ijms-26-03838-s001.zip › ijms-3563122-supplementary.pdf]

Supplementary Materials

Imide polymers with bipolar-type redox-active centers for high-performance aqueous zinc ion battery cathodes and electrochromic materials

Zixuan Liu [1], Yan Li [1], Binhua Mei [1], Jiaxue Liu [1], Haijun Niu [2], Yanjun Hou [*, 1]

[1] *Key Laboratory of Chemistry, Chemical Engineering and Materials, High-Quality Technology Conversion, Heilongjiang Province & School of Chemistry and Chemical Engineering, Heilongjiang University, Harbin, China.*

[2] *Key Laboratory of Functional Inorganic Material Chemistry, Ministry of Education & Department of Macromolecular Science and Engineering, School of Chemistry and Chemical Engineering, Heilongjiang University, Harbin, China.*

* Corresponding author: houyj@hlju.edu.cn

1. Experimental section

1.1 Synthesis of monomer

The process used to create the monomer is depicted in Fig S1. First, p-fluoronitrobenzene (10 mmol, 1.41 g) and aniline (5 mmol, 0.47 g) were reacted with dimethylsulfoxide solution (100 mL) and cesium fluoride (10 mmol, 1.02 g) under catalysis for 48 h at 140 °C. After the reaction was finished, the mixture was poured into iced water while being stirred and filtered to produce 4,4'-dinitrotriphenylamine (yield: 84%, 1.41 g). Next, 4,4'-dinitrotriphenylamine (4.35 mmol, 1.46 g) was reduced in a palladium (1 mmol, 0.11 g) and hydrazine hydrate (10 mL) in ethylene glycol solution (100 mL) at 120 °C for 8 h. Finally, 4,4'-diamino triphenylamine was obtained by filtering the palladium carbon and adding it to iced water while stirring and filtering (yield: 59.3%, 2.58 mmol, 0.71 g). To obtain a white crude product, the crude product was first refined using silica gel column chromatography (petroleum ether:dichloromethane = 1:1), further refined by recrystallization (ether: dichloromethane = 2:1), and then refined further via recrystallization, yielding a white solid in 61% of the sample, ^{1}H NMR (400 MHz, DMSO-d_6) δ 7.09 – 7.00 (m, 2H), 6.80 (d, J = 8.2 Hz, 4H), 6.62 (d, J = 6.9 Hz, 2H), 6.59 (s, 1H), 6.57 – 6.51 (m, 4H), 4.96 (s, 4H).

1.2 Synthesis of polymer

pPMQ and pNTQ: First, 2.5 mmol AQ (0.596 g) was added to 5 mL DMAc and stirred until dissolved. Then, 2.5 mmol PMDA (0.545 g) or NTCDA (0.67 g) was gradually added to the solution in three portions. The mixture formed a poly(amic acid) solution after stirring for 2 h at room temperature. A mixture of 1 mL DMAc and 0.5 mL pyridine dissolved in DMAc was then added to the poly(amic acid) solution and kept at room temperature with constant stirring for 1 h. The temperature was then raised to 120 °C and kept at that temperature for 48 h. The resulting mixture was poured into ice-cold methanol, mixed, stirred, and filtered to produce a brown solid precipitate. Then, it was subjected to Soxhlet extraction with methanol and Dimethylacetamide (DMF) in a 2:1 ratio, and the pPMQT and pNTQT were prepared using a similar procedure with pPMQ and pNTQ. The difference is that the first step involves weighing 4,4-diamino triphenylamine (0.688 g) and AQ (0.596 g), adding them to 5 mL DMAc, and stirring to dissolve them. The rest of the procedure is the same.

2. Calculation methods

2.1 Theoretical specific capacity

The theoretical specific capacity (Q_{Theroy}) of the cathode material can be quantitatively predicted based on the following equation[57]:

$$Q_{Theroy} = \frac{nF}{3.6\mu}$$

Here, n denotes the total number of electrons that can be transferred per active site within a single repeating unit of the polymer. F is the Faraday constant, representing the electric charge carried by one mole of electrons, typically taken as 96,485 $C \cdot mol^{-1}$. M refers to the molar mass of one repeating unit of the polymer material, expressed in $g \cdot mol^{-1}$. The factor 3.6 is a unit conversion coefficient used to convert charge from coulombs (C) to milliampere-hours (mAh).

2.2 Calculation of electron transfer number

For reversible or quasi-reversible redox processes, the peak current of the CV curve is related to the scan rate, diffusion coefficient, electrode area, and the number of electrons transferred. According to the Randles–Sevcik equation:

$$i_p = (2.69 \times 10^5) \cdot n^{3/2} \cdot A \cdot C \cdot D^{1/2} \cdot v^{1/2}$$

Here, i_p denotes the peak current in the CV curve, with units of amperes (A); n is the number of electrons transferred; A represents the surface area of the working electrode (cm^2); C is the concentration of the electroactive species (mol cm^{-3}); D is the diffusion coefficient (cm^2 s^{-1}); and v is the scan rate (V s^{-1}). Based on the Randles–Sevcik equation, n can be derived through the following transformation [58]:

$$n = \left(\frac{i_p}{2.69 \times 10^5 \cdot A \cdot D^{1/2} \cdot C \cdot v^{1/2}}\right)^{2/3}$$

3. Figures and tables

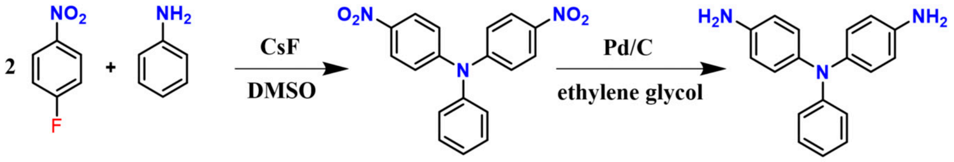

Figure S1. Synthetic reaction pathway of TPA.

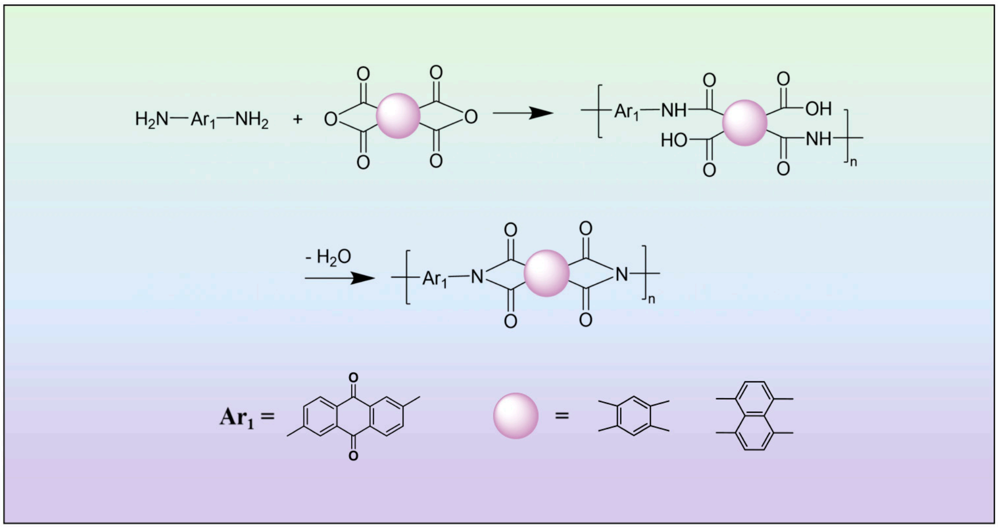

Figure S2. Synthetic routes of pPMQ and pNTQ.

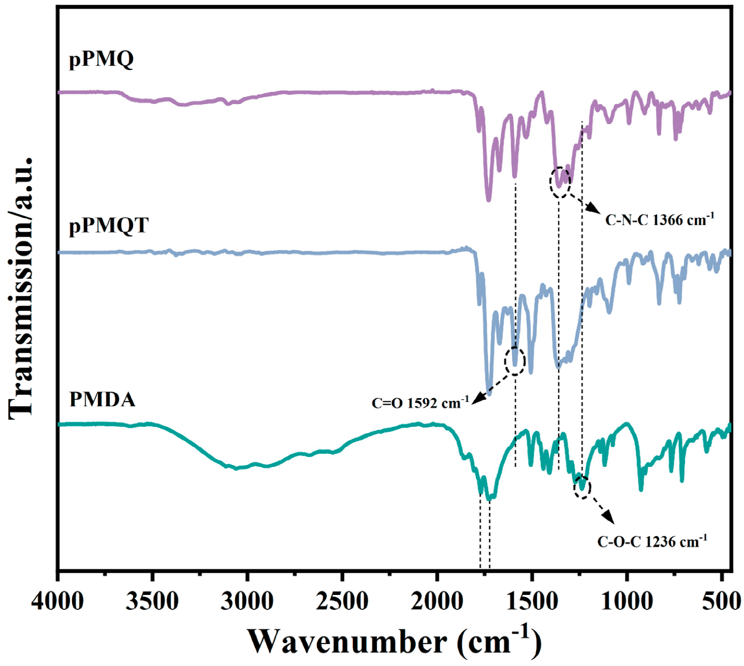

Figure S3. FTIR spectra of pPMQ and pPMQT.

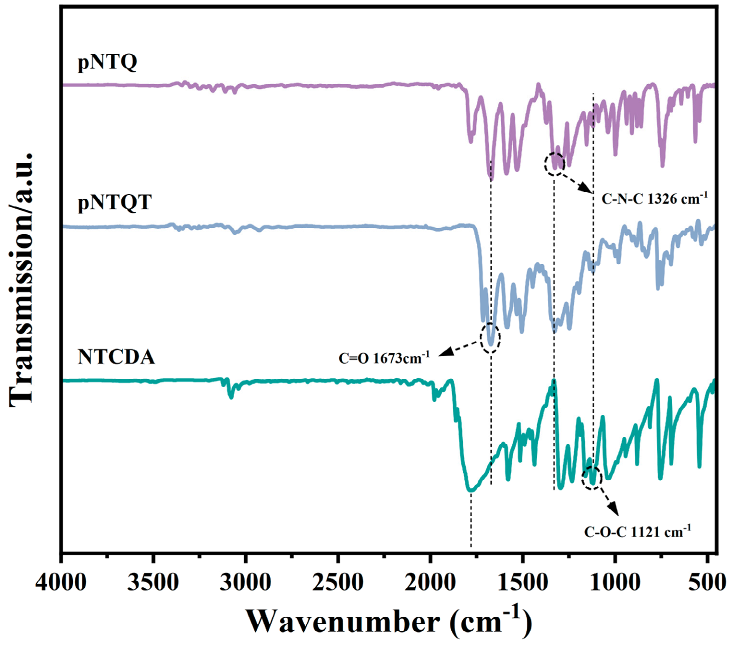

Figure S4. FTIR spectra of pNTQ and pNTQT.

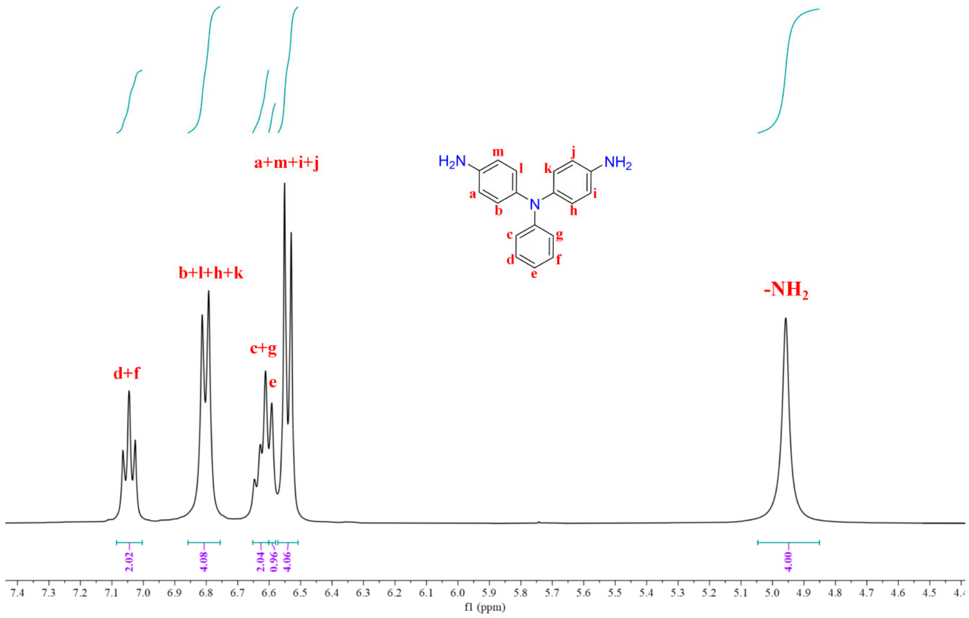

Figure S5. ^{1}H NMR spectrum of 4,4'-diamino triphenylamine.

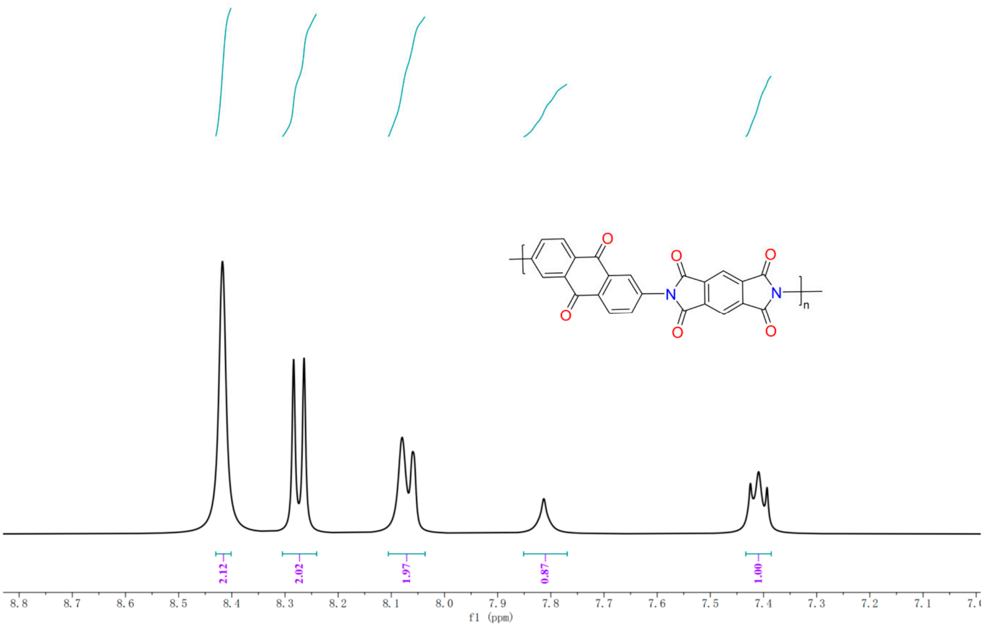

Figure S6. ^{1}H NMR spectrum of pPMQ.

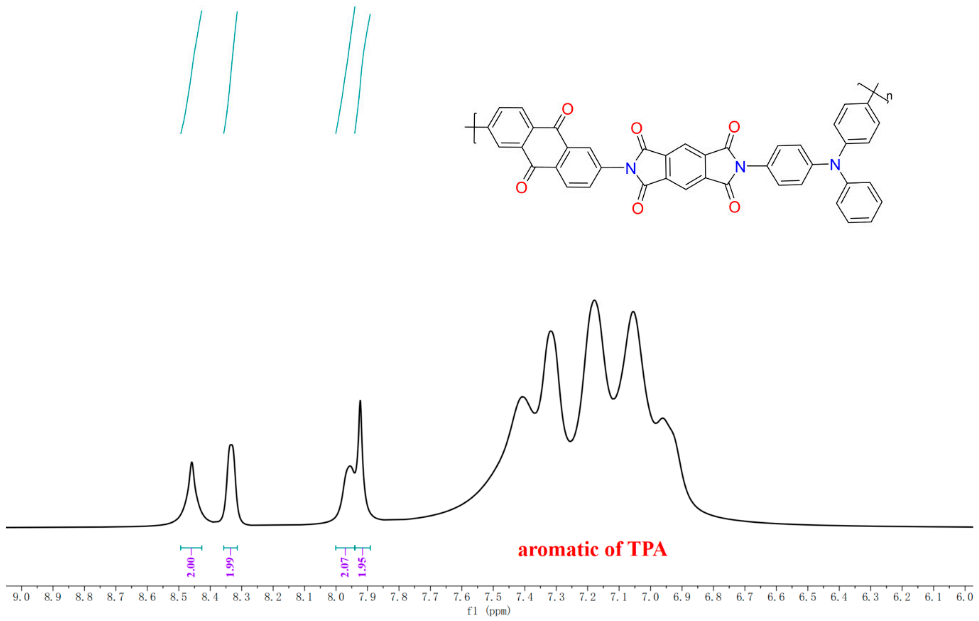

Figure S7. ^{1}H NMR spectrum of pPMQT.

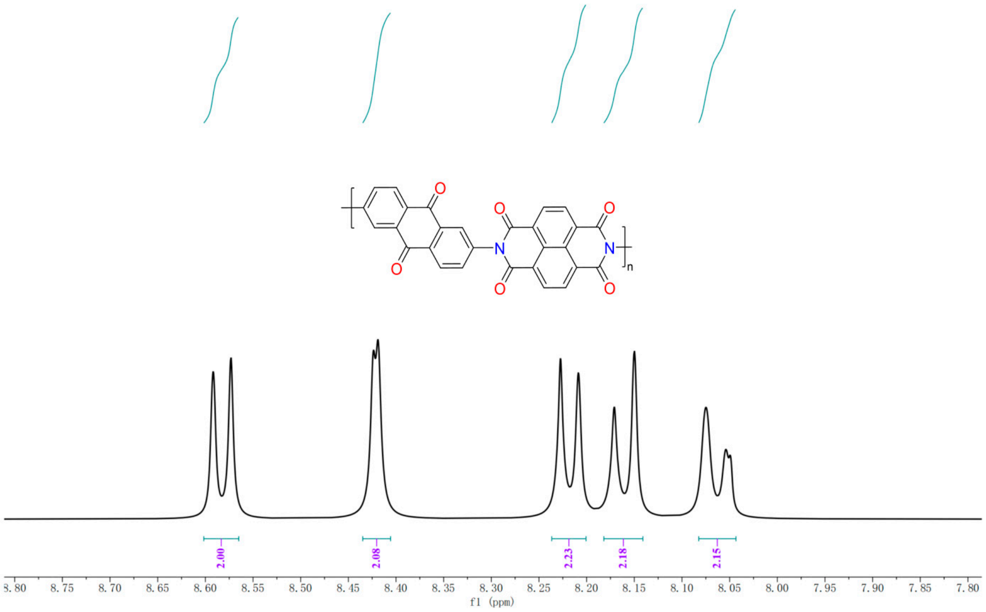

Figure S8. ^{1}H NMR spectrum of pNTQ.

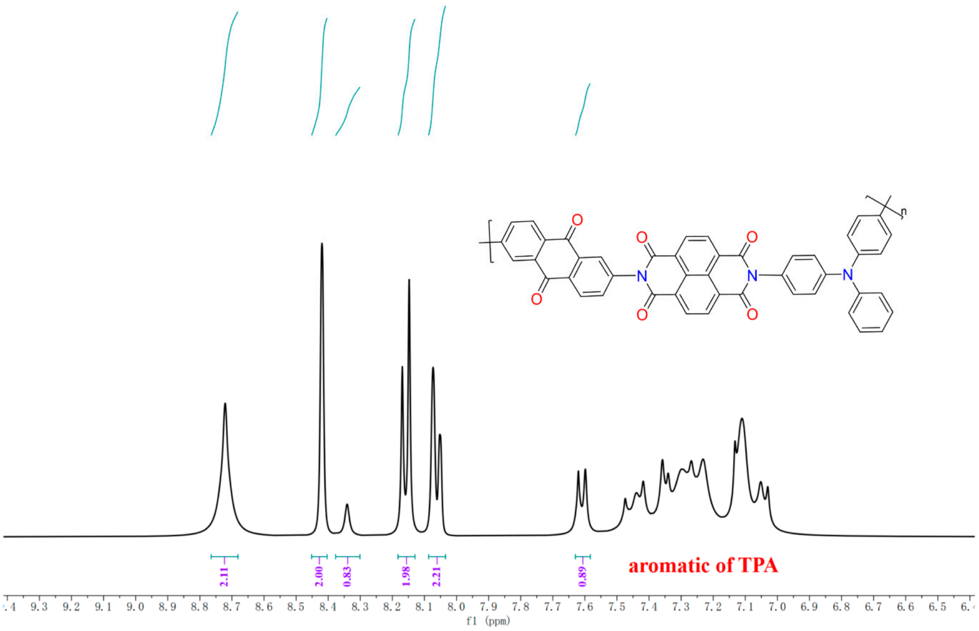

Figure S9. ^{1}H NMR spectrum of pNTQT.

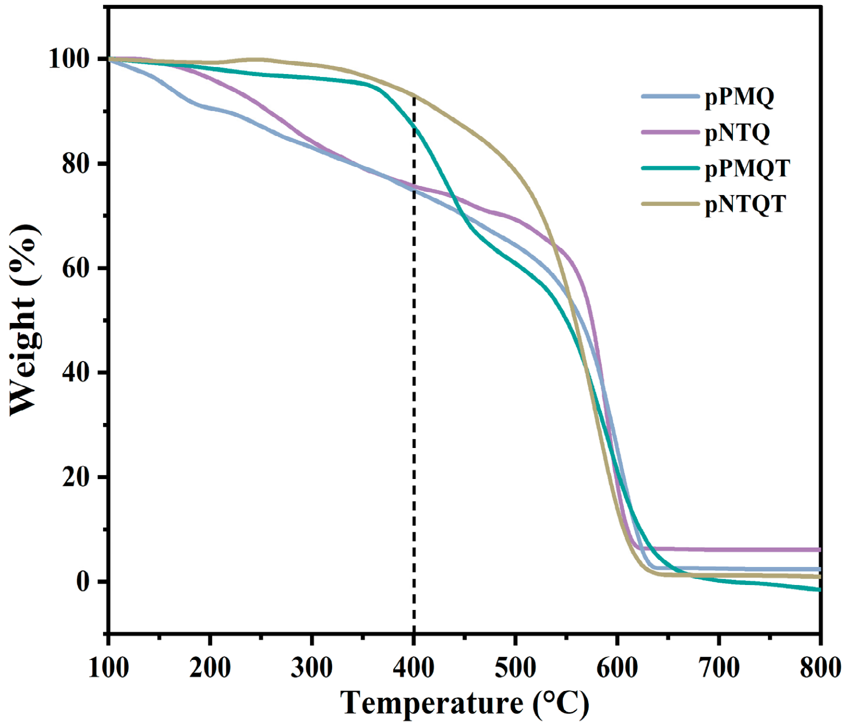

Figure S10. Thermogravimetric curves of pPMQ, pNTQ, pPMQT, and pNTQT.

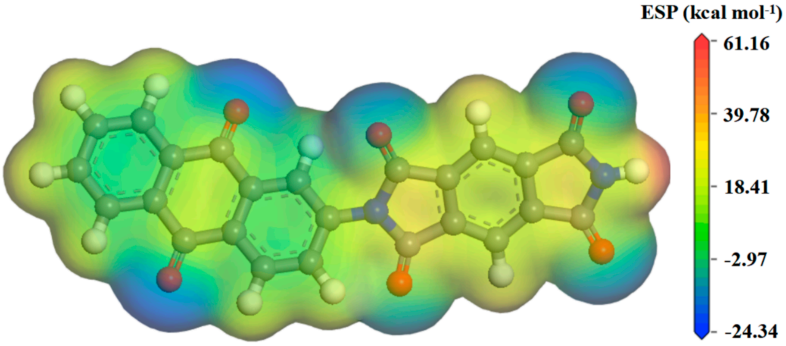

Figure S11. The electrostatic potentials (ESP) of pPMQ.

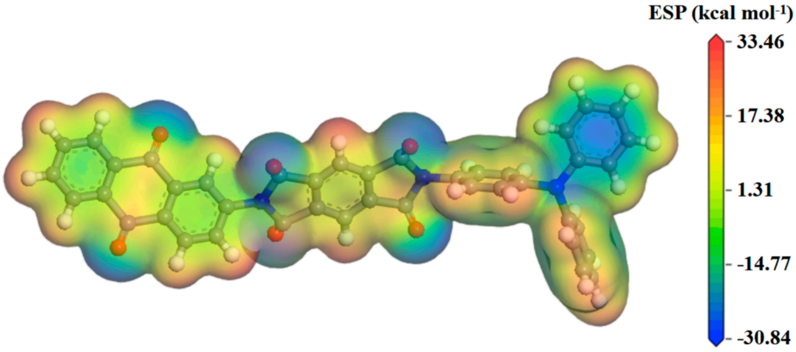

Figure S12. The electrostatic potentials (ESP) of pPMQT.

Table S1. Formal potentials of pPMQT and pNTQT.

Polymer Code	E_{pa}[a]	E_{pc}[b]	E^{0} [c]
pPMQ	0.56	0.32	0.44
pNTQ	0.56	0.46	0.51
pPMQT	0.56/1.15	0.36/1.10	0.46/1.13
pNTQT	0.55/1.14	0.44/1.10	0.50/1.12

[a] The oxidation potential of the polymer .

[b] The reduction potential of the polymer.

[c] E^0 is the formal potential of polymers; $E^0 = (E_{pa} + E_{pc})/2$.

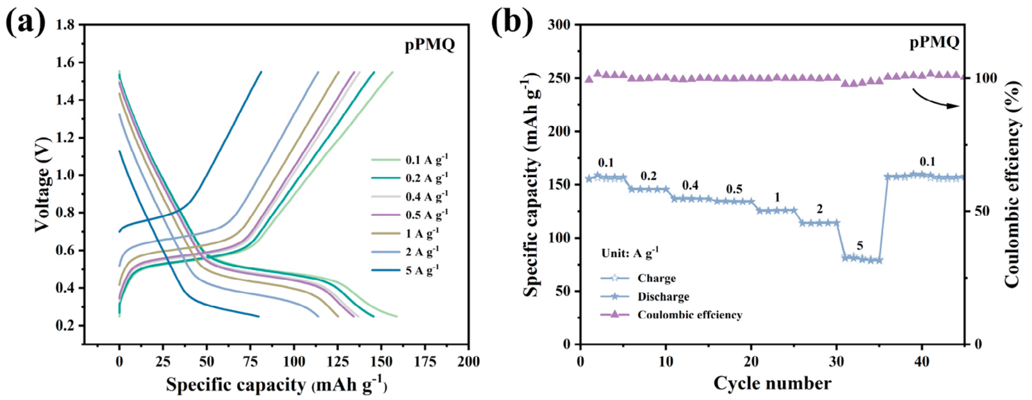

Figure S13. pPMQ with varying current densities: (a) discharge and charge curves and (b) rate performance.

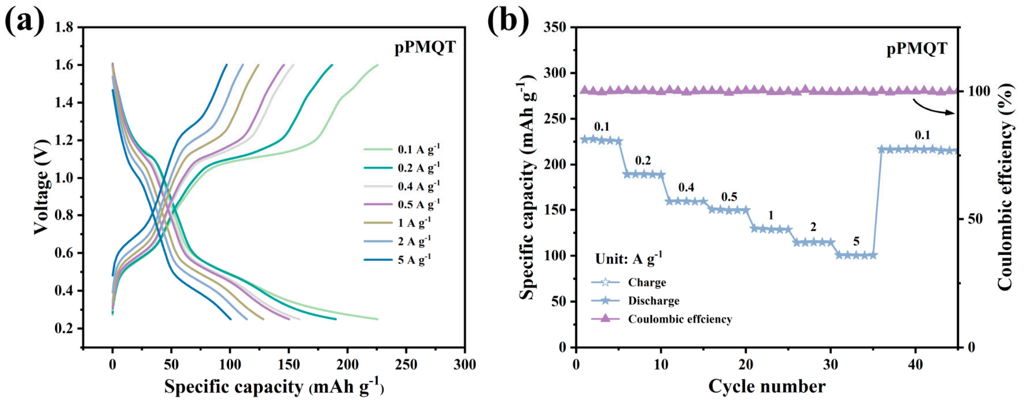

Figure S14. pPMQT with varying current densities: (a) discharge and charge curves and (b) rate performance.

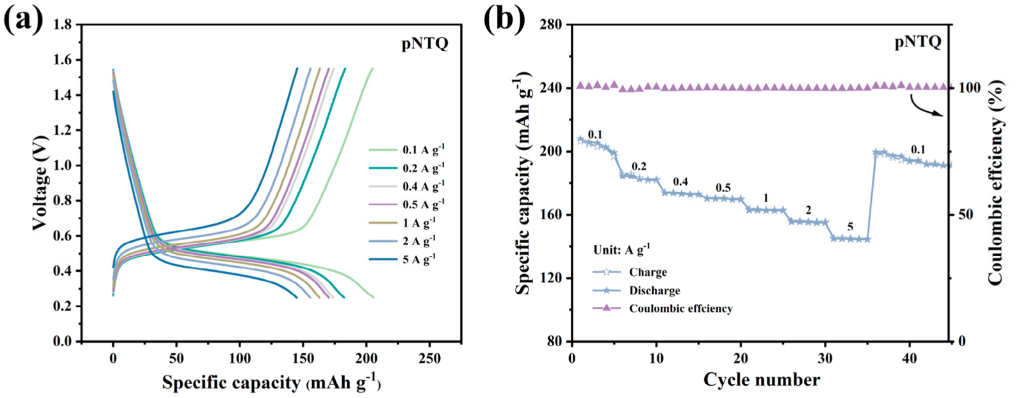

Figure S15. pNTQ with varying current densities: (a) discharge and charge curves and (b) rate performance.

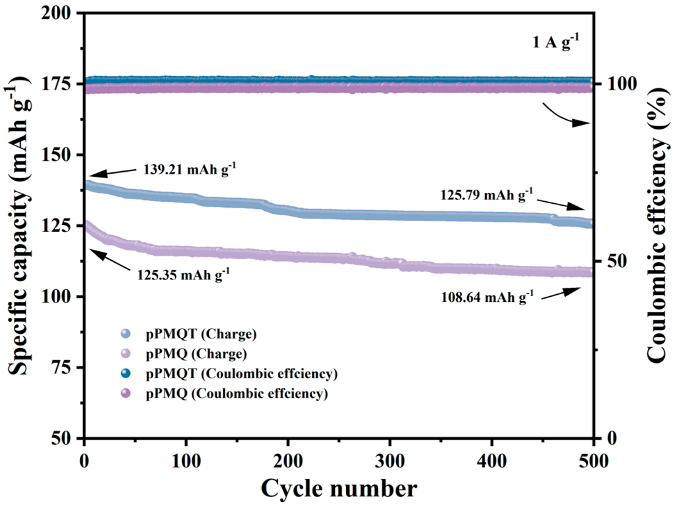

Figure S16. Comparison of long-term cycling stability of pPMQ and pPMQT at 1 A g^{-1} for 500 cycles.

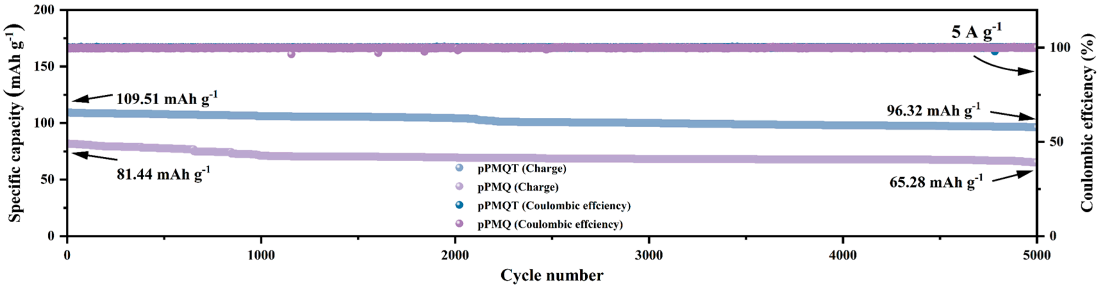

Figure S17. Comparison of long-term cycling stability of pPMQ and pPMQT at 5 A g^{-1} for 5000 cycles.

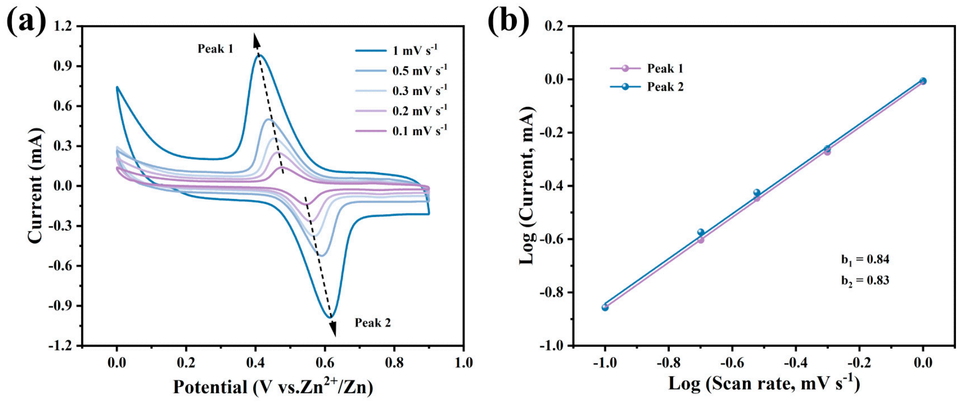

Figure S18. pPMQ half cell (a) CV curves at the scan rates ranging from 0.1 to 1.0 mV s^{-1}. (b) Fitted curves of b values calculated from linear fits of log (i) versus log (v) plots.

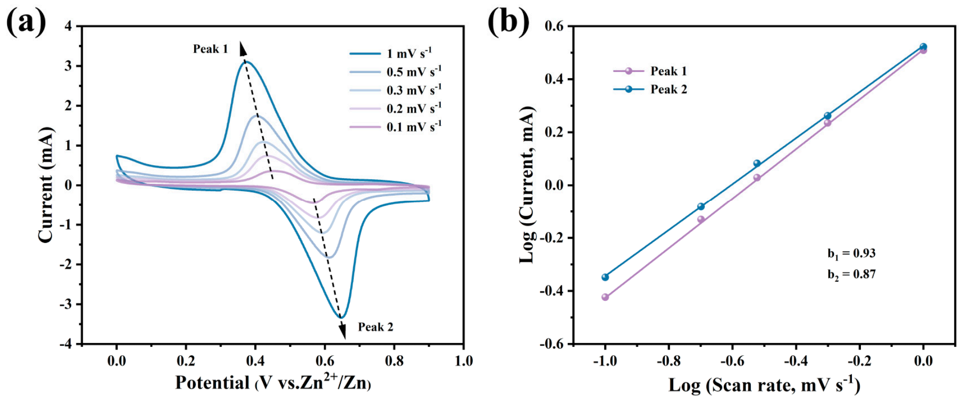

Figure S19. pNTQ half cell (a) CV curves at the scan rates ranging from 0.1 to 1.0 mV s^{-1}. (b) Fitted curves of b values calculated from linear fits of log (i) versus log (v) plots.

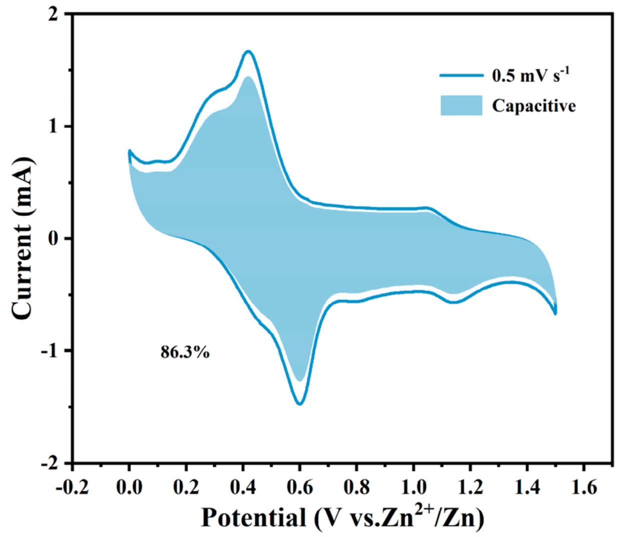

Figure S20. Contribution of capacitive and diffusion control running under 0.5 mV s^{-1} of pNTQT.

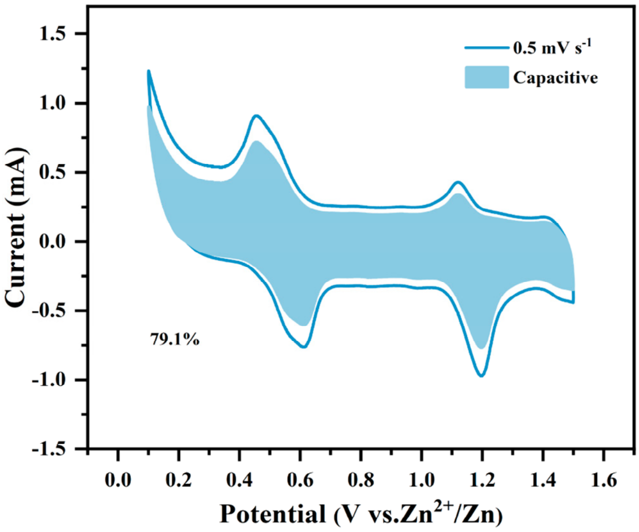

Figure S21. Contribution of capacitive and diffusion control running under 0.5 mV s^{-1} of pPMQT.

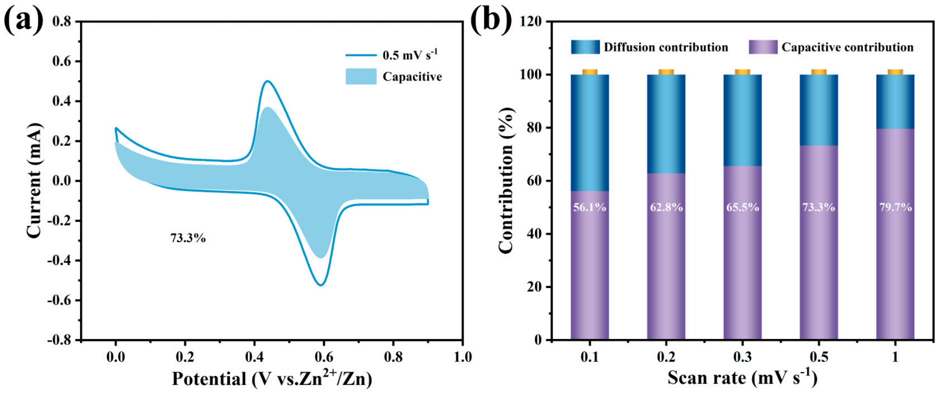

Figure S22. pPMQ half cell (a) Contribution of capacitive and diffusion control running under 0.5 mV s^{-1}. (b) The contributions of capacitive and diffusion control in the scan rate range.

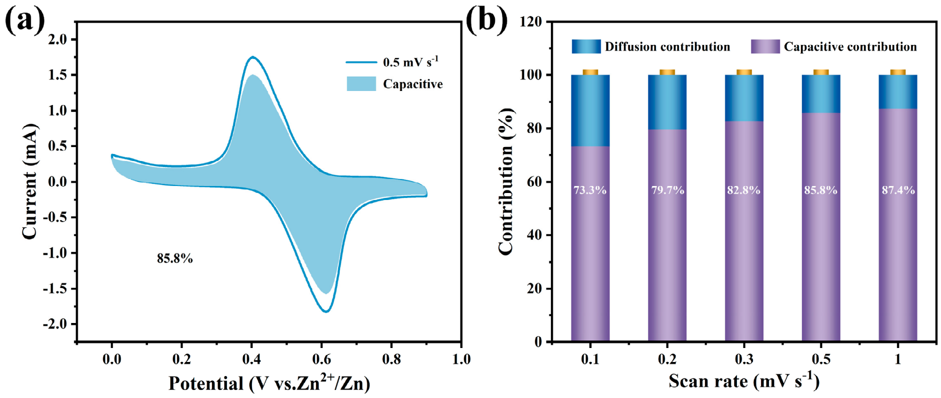

Figure S23. pNTQ half cell. (a) Contribution of capacitive and diffusion control running under 0.5 mV s^{-1}. (b) The contributions of capacitive and diffusion control in the scan rate range.

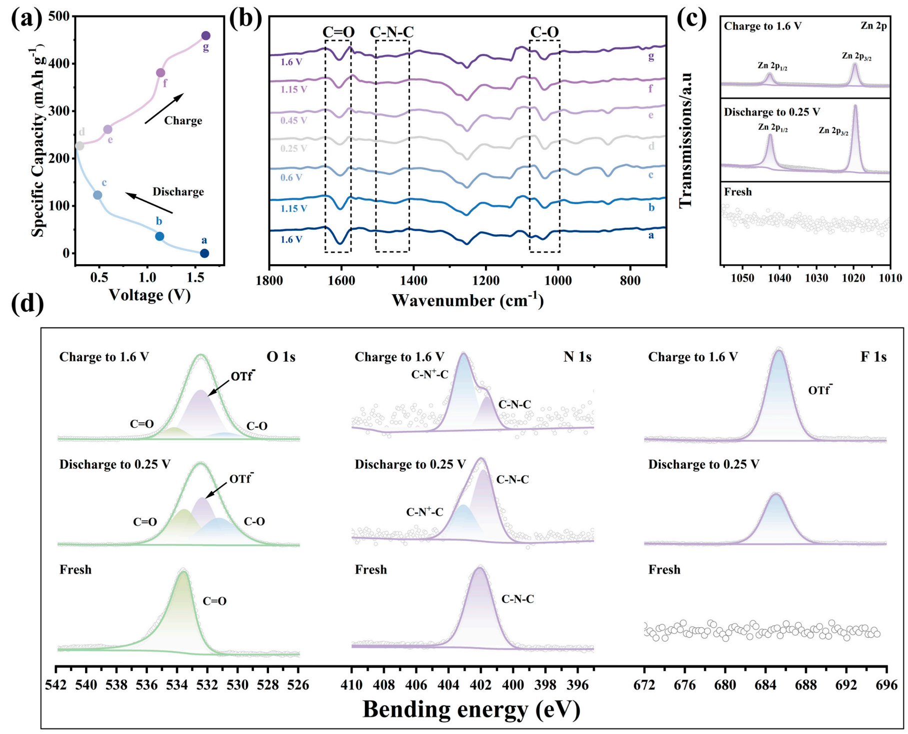

Figure S24. (a) Charge/discharge voltage profiles of pPMQT cathode. (b) Ex situ FT-IR of the pPMQT cathode during the charge/discharge processes. Ex situ XPS spectra of the pPMQT cathode in pristine, fully charged (1.6 V) and fully discharged (0.25 V) states of (c) Zn 2p, (d) O 1s, N 1s, and F 1s spectra are presented on the left, middle, and right, respectively.

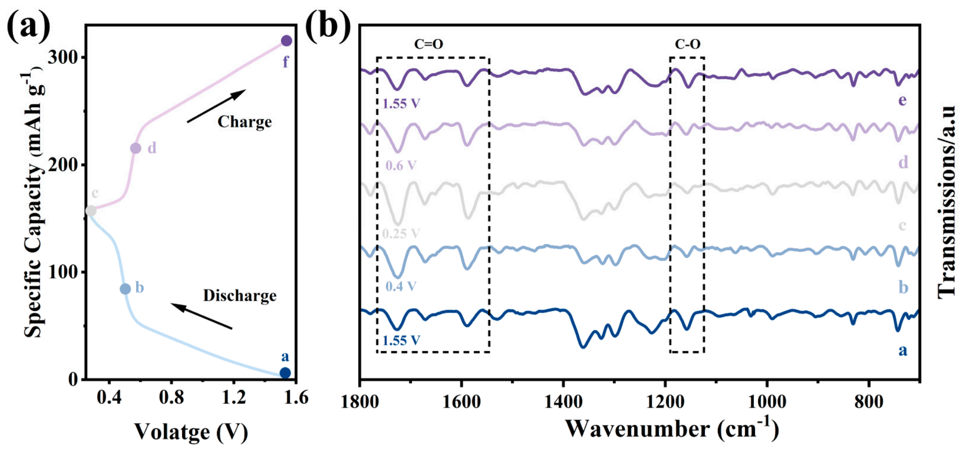

Figure S25. (a) Charge/discharge voltage profiles of pPMQ cathode. (b) Ex situ FTIR of the pPMQ cathode during the charge/discharge processes.

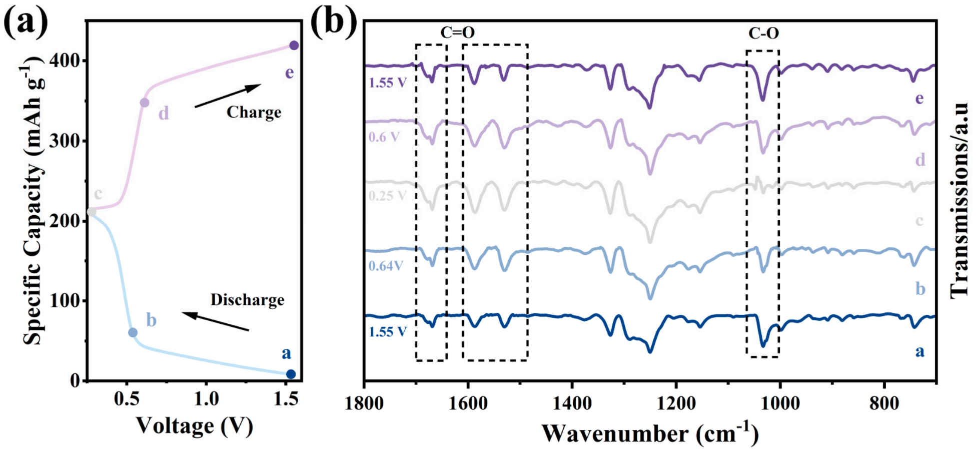

Figure S26. (a) Charge/discharge voltage profiles of pNTQ cathode. (b) Ex situ FTIR of the pNTQ cathode during the charge/discharge processes.

Table S2. The specific atomic concentration data of pPMQT and pNTQT in XPS.

pPMQT	Sample State	Zn 2p Atomic Wt%	N 1s %	O 1s %	F 1s %
	Pristine electrode	-	4.67	9.1	-
	Discharged (0.25 V)	16.48	4.43	20.74	14.97
	Charged (1.6 V)	6.72	2.07	25.34	18.36
**pNTQT**	Pristine electrode	-	4.03	6.9	-
	Discharged (0.25 V)	17.66	4.01	20.25	16.34
	Charged (1.6 V)	6.11	2.04	23.19	20.64

Table S3. Electrochemical properties of pPMQT and pNTQT.

Polymer Code	$\lambda_{onset}^{Abs.}$ [a] (nm)	E_{onset} [b]	$E_{1/2}$ [c] (V)	$E_{electro}$ [d] (eV)			$E_{quantum}$ [e] (eV)		
				E_{HOMO}	E_{LUMO}	Eg	E_{HOMO}	E_{LUMO}	Eg
**pPMQT**	531	0.88	1.06	-5.31	-2.97	2.34	-5.34	-3.41	1.93
**pNTQT**	544	0.81	1.14	-5.24	-2.97	2.27	-5.23	-3.44	1.79

[a] λ_{onset} of polymers in NMP solution (1×10^{-5} mol L^{-1}).

[b] Onset potential (vs. Ag/AgCl) of the polymers was calculated from CV curve in 0.1 M TBAP in ACN.

[c] $E_{1/2}$ of polymers is the average potential of the redox couple peaks.

[d] $E_{LUMO} = E_{HOMO} + E_g$; $E_g = 1240/\lambda_{onset}$ (nm).

[e] Quantum theoretical calculation of the polymers.

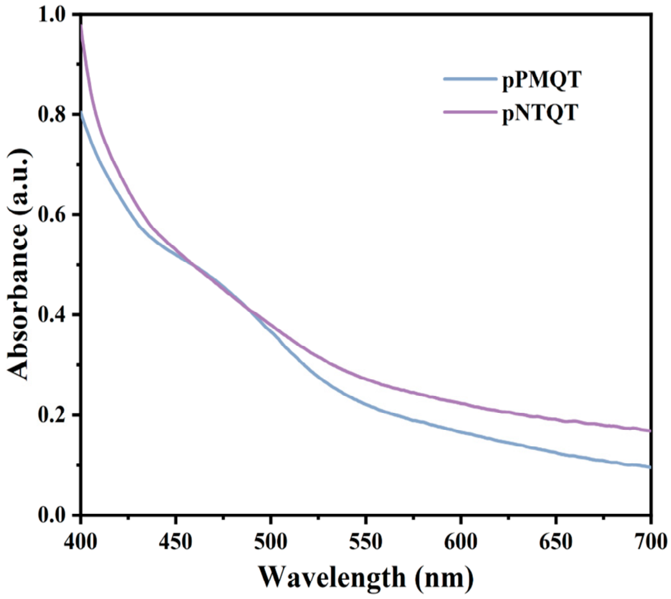

Figure S27. UV–vis absorption spectra of pPMQT and pNTQT in NMP solution.

Table S4. EC properties of pPMQT and pNTQT.

**Polymer Code**	λ^{a} (nm)	ΔT (%)	Response time[b] t_c(s)	t_b(s)	ΔOD[c]	Q_d[d] (mC cm^{-2})	CE[e] (cm^2 mC^{-1})
**pPMQT**	490	32.20	1.7	2.1	0.264	2.27	116.52
**pNTQT**	474	38.49	1.8	2.4	0.322	1.75	182.86

[a] Maximum absorption wavelength of polymer films.

[b] The time for the polymer film to reach 90% of the full-absorption change.

[c] Optical density (ΔOD) = log($T_{blenched}/T_{colored}$), where T_b and T_c are the transmittances in the bleached state and colored state, respectively.

[d] Q_d is ejected charge.

[e] CE = ΔOD/Q_d.

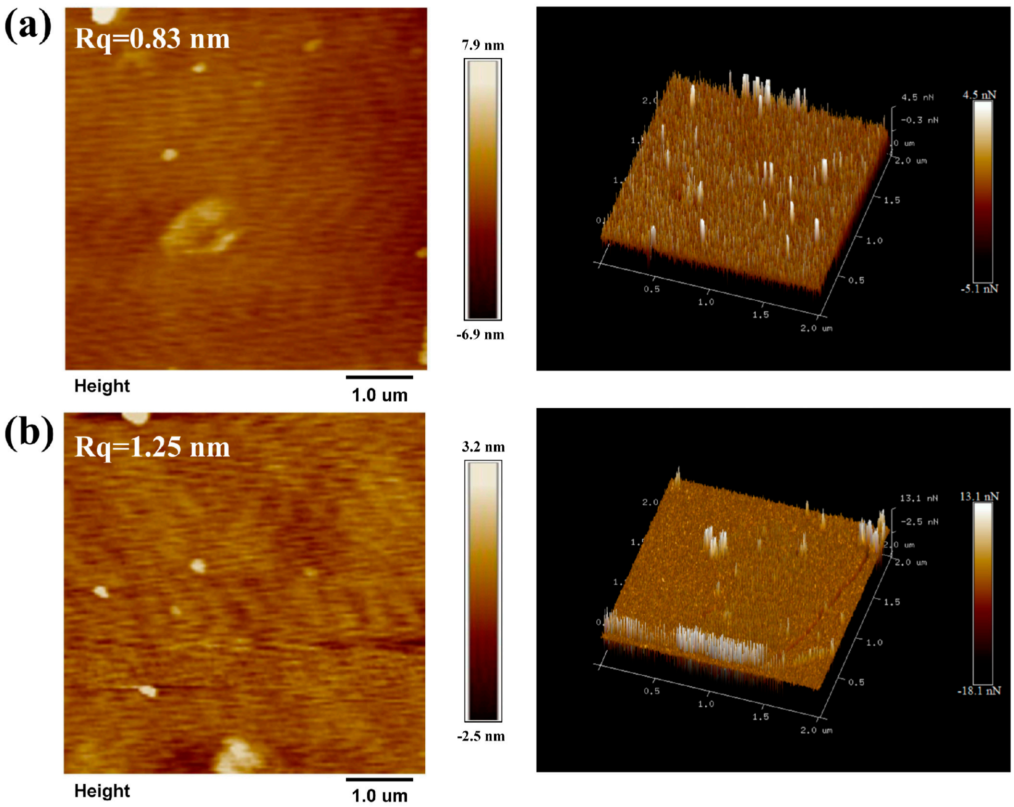

Figure S28. AFM images of pPMQT (a) and pNTQT (b) polymer films.

Reference

57. Li, Z.H.; Tan, J.; Gao, C.Y.; Wang, Y.; Wang, Y.G.; Ye, M.X.; Shen, J.F. Building better aqueous Zn-organic batteries. *Energ Environ. Sci.* **2023**, *16*, 2398-2431.
58. Shitahun, A.; Atlabachew, M.; Aragaw, B.A.; Benor, A.; Metto, M.; Abebe, A. Synthesis, characterization, and application of a novel electrochemical sensor based on poly $[Mn(Chr)_3]Cl_2$/PGE for the determination of ciprofloxacin in pharmaceuticals and urine samples. *Int. J. Electrochem. Sci.* **2025**, *20*, 100937.
